# Assembly of Neuronal Connectivity by Neurotrophic Factors and Leucine-Rich Repeat Proteins

**DOI:** 10.3389/fncel.2016.00199

**Published:** 2016-08-09

**Authors:** Fernanda Ledda, Gustavo Paratcha

**Affiliations:** Division of Molecular and Cellular Neuroscience, Institute of Cell Biology and Neuroscience (IBCN)-CONICET, School of Medicine-University of Buenos Aires (UBA)Buenos Aires, Argentina

**Keywords:** leucine-rich repeat (LRR) proteins, neurotrophic factors, neuronal connectivity, axonal growth and guidance, dendrite development, target innervation

## Abstract

Proper function of the nervous system critically relies on sophisticated neuronal networks interconnected in a highly specific pattern. The architecture of these connections arises from sequential developmental steps such as axonal growth and guidance, dendrite development, target determination, synapse formation and plasticity. Leucine-rich repeat (LRR) transmembrane proteins have been involved in cell-type specific signaling pathways that underlie these developmental processes. The members of this superfamily of proteins execute their functions acting as trans-synaptic cell adhesion molecules involved in target specificity and synapse formation or working in cis as cell-intrinsic modulators of neurotrophic factor receptor trafficking and signaling. In this review, we will focus on novel physiological mechanisms through which LRR proteins regulate neurotrophic factor receptor signaling, highlighting the importance of these modulatory events for proper axonal extension and guidance, tissue innervation and dendrite morphogenesis. Additionally, we discuss few examples linking this set of LRR proteins to neurodevelopmental and psychiatric disorders.

## Introduction

The establishment of a functional nervous system requires connecting neurons into complex neuronal networks with exquisite precision. To achieve this, growing dendrites and axons must navigate toward their target regions, recognize their appropriate target cells and establish specific synaptic contacts with these cells. Along this pathway, dendrites and axons are guided by a group of short- and long-range signals that help them sense and navigate the environment and establish appropriate contacts with specific target cells.

Neurotrophic factors are a group of secreted and diffusible molecules that play a crucial role in the development, survival and maintenance of the peripheral and central nervous system. The two major families of neurotrophic factors are: the neurotrophins and the glial cell line-derived neurotrophic factor (GDNF) family of ligands (GFLs; Reichardt, 2006; Paratcha and Ledda, 2008; Bothwell, 2014; Ibañez and Andressoo, 2016). Neurotrophins constitute a structurally-related family of proteins represented by nerve growth factor (NGF), brain-derived neurotrophic factor (BDNF), neurotrophin 3 (NT3) and NT4, which were first identified as survival factors for developing neurons. Later studies showed that they are pleiotropic molecules that support a variety of functions including the control of axonal growth and guidance, dendrite development and synaptic plasticity via the activation of their cell-surface receptor tyrosine kinase (RTK) TrkA, TrkB and TrkC (Reichardt, 2006; Shmelkov et al., 2010; Mariga et al., 2016). The extracellular domains of the Trk receptors contain two cysteine-rich clusters flanking three leucine-rich repeat (LRR) motives, followed by two immunoglobulin (Ig)-like domains in the juxtamembrane region (Windisch et al., 1995). Binding and deletion studies on TrkA, TrkB and TrkC indicate that one of the Ig-like domains is responsible for neurotrophin binding (Pérez et al., 1995; Urfer et al., 1995; Ultsch et al., 1999), with the leucine-rich domain having a modulatory role in ligand interaction (Windisch et al., 1995).

Neurotrophins are initially synthesized as pro-neurotrophins precursors, which are cleaved to produce the mature protein. Lee et al. (2001) described that pro-neurotrophins can be secreted as precursors (Lee et al., 2001; Hempstead, 2014). While mature neurotrophins bind to Trk receptors and the presence of p75^NTR^ increases the affinity of the Trk receptors for their specific ligands, pro-neurotrophins bind to a p75^NTR^-sortilin receptor complex to induce neuronal cell death during development and pathological conditions (Hempstead, 2014).

GFLs are another family of neurotrophic factors that is composed of GDNF, Neurturin (NRTN), Artemin (ARTN), and Persephin (PSPN). GDNF was originally discovered by its ability to induce the survival of ventral midbrain dopaminergic neurons. Later work showed that GFLs control growth and survival of specific subpopulations of motor neurons and many peripheral neurons, including sympathetic and sensory neurons. GFLs promote these trophic effects acting through two types of receptor subunits, one specialized in ligand binding, represented by the glycosyl-phosphatidylinositol (GPI)-anchored co-receptor, GFRα and another involved in transmembrane signaling, represented by the RTK, Ret or the neural cell adhesion molecule (NCAM; Ibañez and Andressoo, 2016).

Trk and Ret receptors are expressed in specific subpopulations of peripheral and central neurons, where they contribute to the establishment of neuronal connections (Garcès et al., 2000; Patel et al., 2003; Kramer et al., 2006; Wickramasinghe et al., 2008). Although these receptors are expressed in specific subset of neurons, they cannot by themselves explain the complex pattern of neuronal connectivity that characterized the vertebrate nervous system. In recent years, various genetically modified mouse models have demonstrated that Trk and Ret receptors need to be modulated by different proteins to achieve cell type-specific responses to their cognate ligands during circuit development. In this regard, LRR-containing proteins have emerged as relevant partners of neurotrophic factor receptors in specific populations of developing neurons to provide fine tuned control over growth factor signaling.

LRRs are 20–29 amino acid sequence motives present in a number of proteins, which provide structural framework for the formation of protein-protein interactions (Björklund et al., 2006). During the last years, these protein interaction domains have been shown to be critically involved in the control of axonal elongation and navigation, target cell specificity, dendrite development, synapse formation and plasticity. Interestingly, these structural LRR motives are one of the domains more widely maintained across evolution (Chen et al., 2006). The N-terminal region of the repeat consists of a conserved sequence rich in leucine at specified positions (LxxLxLxxNxL, where x is any amino acid). From a structural point of view, each LRR domain is composed of a β-strand connected by a loop region to a α-helix motif roughly parallel to the strand. These repeats commonly fold together to form a curved structure that represent a large binding surface domain, which makes the LRR domain a very effective protein-binding motif (Kobe and Kajava, 2001). Variations in the length and number of repeats affects curvature of the LRR domain and this turn permits interactions with an enormous diversity of ligands. Thus, the LRR domain is not only an efficient but also a very versatile protein-protein interaction domain. The presence of LRR domains in the ectodomain architecture of the Trk neurotrophin receptors anticipated the important roles played by LRR proteins during nervous system development. Many LRR transmembrane proteins are expressed at the synapses where they contribute to target specificity and synapse formation working as trans-synaptic cell adhesion molecules. In addition to this role as synaptic organizers, growing evidence indicates that other LRR domain-containing proteins act in cis modulating cell-surface receptor signaling at different regions and stages of nervous system development.

While flies lack homologs of Trk receptors, Ret is a conserved RTK expressed in the nervous system of vertebrates (Pachnis et al., 1993; Schuchardt et al., 1994; McIlroy et al., 2013) and *Drosophila* (Sugaya et al., 1994; Hahn and Bishop, 2001). Despite being a highly conserved molecule, *Drosophila* Ret (dRet) receptor cannot be activated by their cognate vertebrate GDNF ligands because they are not expressed in flies. However, a recent work has shown novel functions for dRet as a regulator of sensory neuron dendrite growth and patterning, through a mechanism that requires signaling crosstalk with integrins, but does not involve GDNF binding (Soba et al., 2015). Thus, the emergence of neurotrophic factor receptor components and ligands together with the diversification of the LRR proteome across evolution suggest that neurotrophic factor receptor signaling might be considered one of the prerequisites for evolution of complex nervous systems (Dolan et al., 2007).

**Figure 1:**
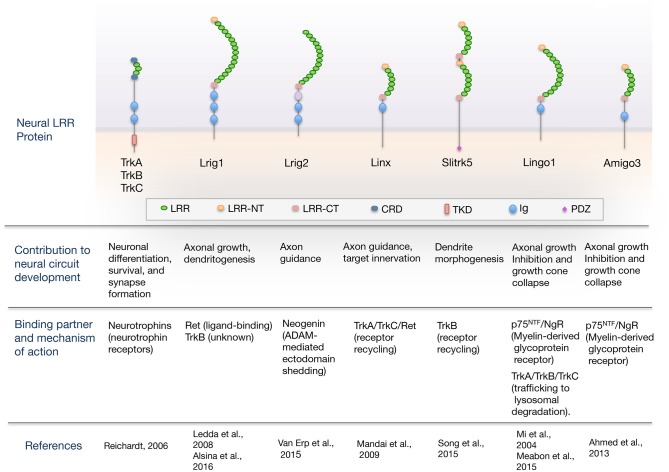
**Overview of the domain organization of leucine-rich repeat (LRR) proteins regulating neural circuit development through their interaction with guidance/neurotrophic factor receptors.** The scheme shows neural LRR proteins discussed in the review, classified by the developmental process that regulate, binding partner and mechanism of action. Domain abbreviations are: Ig, Ig-like domain; LRR, Leucine-rich repeat domain; LRR-NT and LRR-CT, LRR N- and C-terminal domain; CRD, cysteine-rich domain; PDZ, PSD-95/disk-large/zona-occludens-1 domain; TKD, Tyrosine-kinase domain.

Here, we review the current understanding of the role of LRR domain-containing proteins as cell-intrinsic regulators of neurotrophic factor receptor signaling required for proper nervous system development. An overview of the domain organization of LRR proteins regulating neural circuit development through their interaction with neurotrophic factor receptors is shown in Figure 1. For a more comprehensive review that includes other LRR proteins that function as synaptic organizers see the article from de Wit and Ghosh (2014).

## LRR Proteins in the Control of Axonal Growth and Guidance

During the establishment of neuronal circuits, neurons extend axons that navigate over long distances toward their target regions. Along their trajectories, growth cones are guided by the coordinated action of attractive and repulsive cues localized in the extracellular environment. Many evidences indicate that during embryonic development, LRR proteins provide instructive and modulatory signals for axonal navigation, and in this section we discuss the role of LRR proteins in axonal growth, guidance and target innervation.

Trk neurotrophin receptors are a family of LRR proteins that not only promote axonal growth but also have chemotactive effects in the steering direction of growing axons. Various genetically modified mouse models have provided a better understanding of the roles of neurotrophin/Trk signaling in the establishment of neuronal connectivity in the peripheral nervous system. Primary somatosensory neurons located in the dorsal root ganglia (DRG) send axons to the periphery to innervate the skin and muscles, and project central axons to the second-order neurons in the spinal cord. Evidence from different subtypes of somatosensory neurons has revealed a common requirement of target-derived neurotrophins during projection of sensory axons toward their respective central and peripheral targets. Thus, TrkA expressed in cutaneous nociceptive sensory neurons is required for nociceptor innervation of the skin and for the generation of proper axonal projections in the spinal cord (Patel et al., 2003; Guo et al., 2011), and many cutaneous TrkB-positive low-threshold mechanoreceptors require BDNF/TrkB signaling for correct axonal innervation of several types of cutaneous mechanosensory end organs (Perez-Pinera et al., 2008).

In addition to their roles in the peripheral sensory system, Trk receptor signaling is also essential for axonal growth, target innervation and survival of postganglionic sympathetic neurons. Here, intermediate target-derived NT3 (e.g., blood vessels) acts via TrkA to support axonal extension, but not retrograde survival, whereas NGF produced from final targets (e.g., heart) supports not only axonal growth but also survival and gene expression through retrograde signaling. Interestingly, these studies illustrate a model in which a hierarchical neurotrophin signaling cascade coordinates the specificity of sympathetic neuronal innervation (Glebova and Ginty, 2004; Kuruvilla et al., 2004).

Interestingly, in *Drosophila*, there are no canonical homologs of Trk receptors, but two recent studies indicated that the Toll-LRR proteins fulfill this function. In flies, Toll6 and Toll7 have been described as new neurotrophin receptors involved in the control of motor axon targeting and neuronal survival (Lewis et al., 2013; McIlroy et al., 2013). Thus, it will be interesting to study whether the neurotrophins and Toll-like receptor (TLR) protein families interact to control neuronal connectivity also in the vertebrate nervous system.

Several truncated isoforms of TrkB and TrkC, which lack most of the intracellular domain (ICD) have been identified (Reichardt, 2006). Truncated TrkB receptors (TrkB.T1 and TrkB.T2) are splice variants having short cytoplasmic tails lacking the tyrosine kinase domain and are expressed at high levels in the adult brain (Reichardt, 2006). The precise physiological function of these receptors is not completely known, but they seem to act as dominant negative modulators of Trk signaling, capturing neurotrophins, blocking activation of full-length Trk kinases by forming nonproductive heterodimers or even mediating autonomous signaling events directly (Baxter et al., 1997; Hapner et al., 1998; Kryl and Barker, 2000; Esteban et al., 2006). TrkB.T1 is considered the prototype of truncated TrkB receptors. In an effort to understand how the TrkB.T1 receptors contribute to the establishment and development of neural circuits, Liu et al. (2012) demonstrated that androgen-driven expression of the truncated TrkB.T1 in male mammary mesenchyme is required for inhibition of BDNF-induced TrkB signaling in sensory axons. This inhibition of BDNF/TrkB signaling in sensory fibers leads to axonal pruning and generation of the male-type pattern of mammary gland sensory innervation. Therefore, truncated TrkB.T1 receptors direct the sexually dimorphic control of BDNF signaling that determines sensory innervation of the mammary gland (Liu et al., 2012).

Besides having LRR domains, Trk receptors are able to interact with other LRR-containing proteins. Recent data suggest that neurotrophic factor receptors, Trks and Ret physically associate with diverse LRR-containing proteins to modulate their signaling outputs (Ledda et al., 2008; Mandai et al., 2009; Meabon et al., 2015; Song et al., 2015; Alsina et al., 2016).

Linx (alias Islr2) is a LRR transmembrane protein that was identified as a TrkA and Ret signaling component (Mandai et al., 2009). Linx is coexpressed with TrkA and Ret receptors in many developing sensory nociceptive and motor neurons, respectively. Linx interacts with TrkA and Ret receptors and facilitates their activities of axonal growth, guidance and branching. In addition to Linx, several other LRR family members, including Lrig1, Lingo1 and Amigo, are expressed in non-overlapping subset of sensory and motor neurons. This raises the possibility that different LRR proteins encode target specificity by controlling the activity of neurotrophic receptors in select populations of developing neurons (Mandai et al., 2009).

Linx was later described as a key interaxonal signal that guides the extension of central axons independently of its interaction with neurotrophic factor receptors. Linx induces the outgrowth of thalamocortical axons in the ventral forebrain, and mediates thalamortical and corticofugal close interactions at the pallial-subpallial boundary. In this system, Linx do not interact with neurotrophic factor receptors and might cooperatively function with diverse thalamic axon guidance cues, such as slits, netrin-1, neuregulin-1, ephrins, EphB1/2, Sema 3A and Sema 3F (Mandai et al., 2014).

Furthermore, a new role of Linx in ensuring proper retinal axon navigation at the vertebrate optic chiasm has recently been reported (Panza et al., 2015). However, whether the guidance effects of Linx in this system are uncoupled from its neurotrophic function deserves additional investigation.

The LRR and Ig-like domain protein 1 (Lrig1) is another protein that was described as a neurotrophic factor receptor interactor. This molecule belongs to the Lrig family of single-pass transmembrane proteins, which modulate a variety of signaling pathways across evolution. In vertebrates, there are three family members, (Lrig1, Lrig2, and Lrig3), which are defined by closely related extracellular domains with a similar arrangement of 15 LRRs and three Ig-like domains and a cytoplasmic region with no apparent homology to other proteins. Lrig1 is the best functionally-characterized member of the family, which controls the activity of different growth factor RTKs, such as EGFR, ErbB 2–4 and Met, by promoting receptor ubiquitination and degradation (Gur et al., 2004; Laederich et al., 2004; Shattuck et al., 2007; Simion et al., 2014). In 2008, Lrig1 was reported as an endogenous inhibitor of the GDNF receptor Ret (Ledda et al., 2008), able to block GDNF/Ret-induced axonal growth of sympathetic neurons. From a mechanistic point of view, Lrig1 directly interacts with Ret and negatively regulates GDNF/Ret signaling through inhibition of GDNF binding to Ret and recruitment of Ret to lipid raft membrane domains. Despite this, the physiological contribution of Lrig1 for GDNF/Ret-induced axonal growth and peripheral target innervation require additional studies. It is unclear whether Lrig2 and Lrig3 exhibit similar functions to Lrig1 in this system.

Although nothing is known about the role of Lrig2 and Lrig3 on the control of neurotrophic factor receptor signaling, Lrig2 was recently identified as a regulator of axon guidance activities in central neurons. Neural circuit development also requires the precise regulation of guidance receptors at the plasma membrane. Different mechanisms control guidance receptor expression and function at the plasma membrane, including proteolysis by membrane-bound ADAM proteases (Bai and Pfaff, 2011; Kolodkin and Pasterkamp, 2013). Previous work implicated ADAM17 (TACE) in repulsive guidance molecule family member A (RGMa)-Neogenin signaling by showing that this protease cleaves the Neogenin ectodomain in cis and thereby terminates, rather than activates, repulsive Neogenin signaling (Okamura et al., 2011). Ectodomain shedding is crucial for receptor signaling and function, but how this event is regulated in developing neurons has begun to be elucidated. Recent data demonstrated that Lrig2 negatively regulates ectodomain shedding of axon guidance receptors by ADAM17. Lrig2 binds Neogenin, a receptor for RGMs, and prevents premature Neogenin cleavage by ADAM17. RGMa ligands reduce Lrig2-Neogenin complexes, providing ADAM17 access to Neogenin and allowing this protease to induce ectodomain shedding. This regulatory mechanism is required for both the growth cone collapse and the axonal growth inhibitory effects of RGMa-Neogenin signaling (van Erp et al., 2015). Thus, strategies directed to knockdown Lrig2 significantly improved CNS axon regeneration, and identified Lrig2 as a potential therapeutic target for promoting regeneration of injured axons.

Interestingly, it has been reported that mutations in Lrig2 cause urofacial syndrome (UFS), an autosomal-recessive disease characterized by congenital urinary bladder dysfunction associated with a significant risk of kidney failure and abnormal facial expression. Although the mechanism underlying this effect is still unknown, the evidence indicates that Lrig2 is required for normal autonomic innervation of the urinary tract (Stuart et al., 2013; Woolf et al., 2014). In addition to this effect of Lrig2, sensory innervation of the cochlea has been observed to be disrupted in Lrig1:Lrig2 double-mutant mice, indicating that Lrig1 and Lrig2 exert complementary functions during inner ear innervation. Together these findings uncover novel roles of Lrig2 in the peripheral nervous system, perhaps functioning as modulators of neurotrophic factor signaling pathways (Del Rio et al., 2013).

The Slitrk family includes six brain-specific transmembrane proteins (Slitrk1–6) that contain extracellular LRR domains homologous to the axon guidance molecule Slit and intracellular C-terminal tyrosine residues with sequences homologous to the Trk molecules. In particular, Slitrk6 promotes the survival and innervation of sensory neurons in the inner ear, at least in part by modulating neurotrophin–Trk receptor signaling. Interestinlgy, *BDNF* and *NT3* mRNA levels, as well as their cognate receptors TrkB and TrkC, were downregulated in the inner ear of Slitrk6-knockout mice, indicating that Slitrk6 acts as a positive regulator of TrkB and TrkC signaling (Katayama et al., 2009).

Lingo1 is a transmembrane LRR protein that has been reported to influence axonal growth through two different mechanisms, one involving its interaction with p75^NTR^ and other partnering with Trk neurotrophin receptors (Mi et al., 2004; Meabon et al., 2015). Initially, Lingo1 was identified as a crucial component of the p75^NTR^/NgR1 (Nogo receptor, a GPI-anchored LRR protein) complex mediating axonal growth cone collapse (Mi et al., 2004). Axonal regeneration in the injured adult CNS is inhibited by myelin-derived inhibitory molecules (Nogo, MAG and OMgp), which bind to NgR1 and trigger growth cone collapse through the Rho GTPase pathway by associating with the two transmembrane co-receptors p75^NTR^ and Lingo1 (Wang et al., 2002; Filbin, 2003; Mi et al., 2004). More recent findings indicate a novel role for Lingo1 in the modulation of Trk RTK activity (Fu et al., 2010; Meabon et al., 2015). Here, the suggested model indicates that Lingo1 encounters activated Trk receptors in endosomes and this interaction leads Trks away from the recycling pathway and directs them into endolysosomal pathway for its degradation. Thus, Lingo1 acts as a negative regulator of Trk neurotrophin receptor signaling.

A recent study showed that another member of the superfamily of LRR-containing proteins, Amphoterin-induced gene and open reading frame 3 (Amigo3), represents a novel and alternative p75^NTR^/NgR co-receptor that mediates myelin-associated axonal growth inhibition in the acute phase of adult CNS injury (Ahmed et al., 2013). Thus, blocking Amigo3 immediately after CNS injury might provide a very effective therapeutic strategy for promoting axon regeneration when combined with neurotrophic factor administration. Amigo3 belongs to a family of transmembrane proteins with six LRR motives and a single Ig-like domain, which includes two other members. The expression of Amigo members is enriched in the nervous system. The three members of the Amigo family exhibit both homophilic and heterophilic binding activity that may facilitate neuronal growth functioning as adhesion molecules.

## LRR Proteins in the Control of Dendritic Growth and Morphology

Dendrite size and morphology are key determinants of the functional properties of neurons, and many neurodevelopmental and psychiatric disorders are due primarily to structural abnormalities of dendrites and their connections (Kulkarni and Firestein, 2012). Neurotrophin signaling through Trk receptors is one of the most studied molecular complexes that promote growth, branching and spine density of developing dendrites in different brain areas (McAllister et al., 1995; Imamura and Greer, 2009; Kwon et al., 2011; Lazo et al., 2013; Joo et al., 2014). In particular, BDNF and its receptor TrkB have been described to be required for dendritic growth and spine formation in different brain regions including the cortex, hippocampus and striatum. Target deletion of BDNF in mice from excitatory CNS neurons resulted in deficits in dendritic growth and spine density in different brain regions (Rauskolb et al., 2010).

Moreover, NT4 has also been described as a dendritogenic factor for cortical pyramidal cells and interneurons (Wirth et al., 2003). Recent studies revealed that NT3/TrkC signaling modulates dendritic complexity of Purkinje cerebellar neurons *in vivo* (Joo et al., 2014). Intriguingly, the authors described that TrkC-deficient Purkinje neurons have reduced complexity, which is rescued by removal of NT3.

Although neurotrophins and their receptors have essential roles in dendrite patterning, how a limited repertoire of neurotrophins and Trk receptors promote a diverse array of dendritic patterns is a question that has recently begun to be answered. In this sense, recent studies have identified key cell-intrinsic regulators of the diverse array of dendrite morphologies promoted by neurotrophins in the CNS. In one of these studies, Slitrk5 has been identified as a novel TrkB receptor interactor. Although all members of Slitrk family expressed in the postsynaptic compartment induce presynaptic differentiation by trans-synaptic interaction with Protein Tyrosine Phosphatases (PRPs) located in the presynaptic side, Slitrk5 has emerged as a TrkB receptor component that facilitates BDNF-induced TrkB signaling required to dendrite morphogenesis of striatal GABAergic neurons. Upon BDNF stimulation, Slitrk5 forms a physical complex with TrkB through their LRR domains and directs TrkB receptor to Rab11-positive recycling endosomes promoting neurotrophin signaling. In this context, Slitrk5-null mice exhibit reduced striatal volume, decreased dendrite complexity and affected basal ganglia circuitry and function, similar to the phenotype reported in the striatum of mice with genetic deficiencies in BDNF and TrkB signaling (Song et al., 2015).

Recently, the nervous system-enriched LRR protein Lrig1 was identified as a physiological regulator of proximal hippocampal dendrite arborization and BDNF signaling. Lrig1-deficient animals show an increase in primary dendrite formation and dendritic branching of hippocampal pyramidal neurons, two phenotypes that resemble the effect of BDNF on these neurons. In addition to restrict dendrite morphogenesis, overexpression of Lrig1 also blocked the formation of dendritic spines induced by BDNF in these neurons. Despite this, the exact mechanism through which Lrig1 controls BDNF signaling is not completely known, a physical interaction between TrkB and Lrig1 seems to be required to inhibit receptor activation and BDNF signaling (Alsina et al., 2016). During development, different neuronal domains encounter similar environmental factors. However, intrinsic modulators, located within specific domains of the neurons, control the cellular interpretation of these extrinsic cues, thereby allowing neurons to generate distinct morphologies. Interestingly, Lrig1 is located in the soma and extends out into the apical dendrites of hippocampal pyramidal neurons, controlling BDNF signaling in this neuronal domain. In agreement with this, ablation of Lrig1 preferentially increases the proximal complexity of the apical dendrites of hippocampal CA1–CA3 pyramidal neurons, revealing a novel molecular mechanism involved in basal vs. apical dendrite morphogenesis.

Together, these findings establish that Slitrk5 and Lrig1 are essential molecules linking TrkB signaling to dendrite development in different brain areas.

## LRR Proteins and Disorders of the Nervous System

Given the critical role played by genes encoding LRR proteins in the organization and function of neural circuits, it seems likely that mutations in genes encoding LRR proteins or in their binding partners could compromise axon guidance, dendrite arborization, synapse development and lead to a range of neurodevelopmental and psychiatric disorders. LRR proteins have been directly linked to different human brain disorders, including autism, schizophrenia, obsessive-compulsive disorders (OCD), epilepsy, essential tremor (ET), Alzheimer’s (AD) and Parkinson’s disease (PD; de Wit et al., 2011; Ko, 2012; see Table 1). Here, we focus on those LRR proteins that modulate signaling by neurotrophic factors and that are discussed in this review.

**Table 1:** **Leucine-rich repeat (LRR) proteins implicated in neurodegenerative, neurodevelopmental and psychiatric disorders**.

LRR proteins	Behavioral defects/Disorder	Reference
TrkA	Congenital insensitivity to pain with anhidrosis (CIPA)	Indo (2014)
	Schizophrenia	Van Schijndel et al. (2011)
TrkB	Alzheimer’s disease	Chen et al. (2008)
	Autism spectrum disorder (ASD)	Chandley et al. (2015)
	Huntington’s disease	Plotkin et al. (2014) and Nguyen et al. (2016)
	Bipolar disease	Dmitrzak-Weglarz et al. (2008)
	Schizophrenia and mood disorders	Ray et al. (2011); Ray et al. (2014)
	Epilepsy	Liu et al. (2013)
Lingo1	Parkinson’s disease and essential tremor	Inoue et al. (2007); Kuo et al. (2013) and Delay et al. (2014)
	Glaucoma	Fu et al. (2008); Fu et al. (2010)
	Schizophrenia	Fernandez-Enright et al. (2014)
Lrig1	Abnormal social interaction	Alsina et al. (2016)
Lrig2	Urofacial syndrome (UFS)/congenital urinary bladder	Stuart et al. (2013) and Woolf et al. (2014)
Slitrk1	Tourette’s syndrome	Abelson et al. (2005)
Slitrk5	Obsessive-compulsive disorder (OCD). Anxiety	Shmelkov et al. (2010)

*The table includes those LRR proteins covered in this review. Some behavioral phenotypes obtained in deficient mice are also mentioned*.

Considering the significant heritability of many neurodevelopmental and psychiatric disorders, recent efforts have focused on the search for susceptibility genes for these diseases. A recent study has revealed that Slitrk5-deficient mice show increased anxiety-like and excessive self-grooming behaviors, characteristic of obsessive-compulsive-like behavior (Shmelkov et al., 2010). Treatment with chronic fluoxetine, a selective serotonin reuptake inhibitor used to treat depression and OCD, alleviated the excessive grooming behavior observed in Sllitrk5 null mice. Further analysis of Slitrk5 knockout mice indicates that corticostriatal circuitry, which is implicated in the pathogenesis of OCD, is specifically affected by deletion of Slitrk5. Moreover, Slitrk5 deficiency reduces dendrite complexity of striatal medium spiny neurons and impairs corticostriatal neurotransmission, an effect that may be due to lower expression of glutamate receptors in the striatum (Shmelkov et al., 2010). Together this genetic evidence clearly supports a role of Slitrk5 for the development of the neural circuits associated with anxiety disorders and establishes a new mouse model of OCD-like behaviors. Interestingly, Slitrk1-deficient mice also display elevated anxiety-like behavior characteristics of OCD (Katayama et al., 2010). Moreover, Slitrk1 has also been identified as a candidate gene for Tourette’s syndrome (TS), a psychiatric disorder characterized by involuntary physical and vocal tics (Abelson et al., 2005).

Mental retardation and autism spectrum disorders are genetic diseases often associated with overgrowth or lack of dendrite pruning during development and characterized by impaired sociability and altered social phenotypes. Recently, it was reported that Lrig1-deficient mice exhibit hippocampal dendritic abnormalities that correlate with deficits in social interaction (Alsina et al., 2016). Intriguingly, these findings raise the possibility that Lrig1 dysfunction may contribute to different cognitive and developmental brain disorders.

Several studies revealed detrimental roles of Lingo1 during nervous system development (Mi et al., 2009; Andrews and Fernandez-Enright, 2015). Lingo1 was first implicated in PD, when it was discovered that Lingo1 is upregulated in the substantia nigra of post mortem PD brains and in animal models of PD after neurotoxic lesions of the midbrain dopaminergic neurons (Inoue et al., 2007). In one of these studies, Inoue et al. (2007) demonstrated that Lingo1 antagonists improve dopamine neuron survival, growth, and function using *in vivo* models of PD. This neuroprotective effect involves the activation of the EGFR/PI3K/Akt signaling pathway through a direct inhibition of Lingo1’s binding to the EGFR (Inoue et al., 2007).

ET is the most prevalent adult-onset movement disorder. Higher levels of Lingo1 protein have been detected in cerebellum of ET patients (Kuo et al., 2013; Delay et al., 2014). It is hypothesized that these changes are due to post-transcriptional alterations, because not mRNA alterations were detected. Therefore, the downregulation of Lingo1 in the cerebellum is a potential disease-modifying therapeutic target for this pathology. In agreement with this, Lingo1 has already received attention concerning its potential as drug target of interest for treatment of PD (Inoue et al., 2007), and other pathologies of the nervous system (Ji et al., 2006; Satoh et al., 2007).

Glaucoma is another neurodegenerative disease characterized by abnormally high intraocular pressure and blindness due to retinal ganglion cell (RGC) death. Both Lingo1 and NgR were found to be upregulated in RGCs in an ocular hypertension paradigm in rats (Fu et al., 2008, 2010). Blocking Lingo1 function, either using Lingo1-Fc or antibodies against Lingo1, has been shown to not only reduce the number of RGCs that were lost after ocular hypertension, but also promote the survival of RGCs following optic nerve injury (Fu et al., 2008, 2010). The mechanism through which Lingo1 inhibitors induce neuroprotection involves the RhoA and PI3K/Akt signaling pathways. Lingo1 is able to form a complex with TrkB following ocular hypertension, and it was observed that Lingo1 antagonists caused higher TrkB activation than BDNF alone, leading to the survival of RGCs (Fu et al., 2009, 2010).

Recently, Fernandez-Enright et al. (2014) have provided the first evidence of an implication of Lingo1 in schizophrenia, by observing alterations in the expression profiles of Lingo1 and their signaling partner proteins in the dorsolateral prefrontal cortex and CA1–CA3 hippocampal areas of post-mortem schizophrenia brains, two regions highly disrupted in this pathology.

Finally, altered BDNF/TrkB signaling has been implicated in the pathophysiology of neurodegenerative and psychiatric disorders including, AD, PD, HD, bipolar disease and epilepsy (Chen et al., 2008; Dmitrzak-Weglarz et al., 2008; Gupta et al., 2013; Liu et al., 2013; Plotkin et al., 2014; Chandley et al., 2015; Mariga et al., 2016; Nguyen et al., 2016). Levels of TrkB mRNA were reported to decrease in the hippocampus of patients suffering from schizophrenia and/or mood disorders (Ray et al., 2011, 2014) and TrkA polymorphism has also been associated with schizophrenia (Van Schijndel et al., 2011). Considering the regulatory role of different LRR proteins on Trk signaling, it becomes important to understand the role of these modulators in the neurodegenerative and psychiatric disorders associated with neurotrophins and Trks.

Finally, genetic evidence also links some LRR proteins with congenital disorders of the nervous system. Thus, Lrig2 has been associated with congenital urinary bladder dysfunction, a disease characterized by abnormal innervation of the urinary tract (Stuart et al., 2013; Woolf et al., 2014). In addition, loss of function mutations in TrkA have been associated with congenital insentivity to pain with anhidrosis (CIPA), an autosomal genetic disorder characterized by the absence of NGF-dependent primary afferent fibers and sympathetic postganglionic neurons (Indo, 2014).

Thus, the modulation of neurotrophic factor receptor signaling represents a powerful therapeutic approach for reversing neuronal dysfunctions associated with neurodegenerative, neurodevelopmental and psychiatric disorders.

## Concluding Remarks

More than 20 years after the discovery of neurotrophic factor receptors, recent findings continue providing novel receptor modulators/components capable to execute complex regulatory mechanisms of receptor signaling that diversify neurotrophic function. These studies establish that engagement of neurotrophic receptors with specific LRR proteins represents a general mechanism that explains how neurons within a particular region of the nervous system expand the repertoire of neurotrophic factor signaling outputs during circuit development. We are now beginning at understanding the mechanistic details through which LRR proteins regulate guidance/neurotrophic factor receptor signaling. The evidence obtained so far indicate that these proteins can modulate receptor activity in opposite ways, inhibiting or promoting receptor activation through different mechanisms such as ligand binding, receptor ubiquitination, shedding, and trafficking (Ledda et al., 2008; Simion et al., 2014; Meabon et al., 2015; Song et al., 2015; van Erp et al., 2015).

Many LRR proteins have multiple binding partners across the synapses, in trans, and as neurotrophin receptor interactors functioning in cis. Despite these advances, several key questions remain unanswered. For instance, it will be important to fully characterize these protein interaction networks, identify the molecular domains that govern these interactions, and determine the physiological contribution of these proteins for nervous system development and function.

Although several questions remain to be addressed, an increased understanding of the roles of LRR proteins promises to give insights into the many neurodevelopmental and psychiatric disorders typified by alterations in neural circuitry, and may aid to achieve effective therapies to cure these neurological diseases.

Neurotrophic factors have emerged as promising disease-modifying molecules for neurological disorders and regeneration of damaged axons, and some of them are currently being used in clinical trials. However, they had limited success in the treatment of neurodegenerative diseases due to pharmacokinetic and delivery issues. It appears likely that many of these LRR proteins represent new potential therapeutic targets for these disorders. Thus, targeting endogenous regulators/components of neurotrophic factor receptor signaling, such as Lrig1, Slitrk5, Lingo1 and Amigo3, might be of clinical interest, maximizing the therapeutic power of neurotrophic factors on injured neurons in a region-specific manner.

## Author Contributions

FL and GP wrote the manuscript.

## Conflict of Interest Statement

The authors declare that the research was conducted in the absence of any commercial or financial relationships that could be construed as a potential conflict of interest.
